# A Smart Crop Water Stress Index-Based IoT Solution for Precision Irrigation of Wine Grape

**DOI:** 10.3390/s24010025

**Published:** 2023-12-20

**Authors:** Fernando Fuentes-Peñailillo, Samuel Ortega-Farías, Cesar Acevedo-Opazo, Marco Rivera, Miguel Araya-Alman

**Affiliations:** 1 Instituto de Investigación Interdisciplinaria (I3), Vicerrectoría Académica (VRA), Universidad de Talca, Talca 3460000, Chile; ffuentesp@utalca.cl; 2 Research and Extension Center for Irrigation and Agroclimatology (CITRA) and Research Program on Adaptation of Agriculture to Climate Change (A2C2), Faculty of Agricultural Science, University of Talca, Talca 3460000, Chile; 3 Power Electronics, Machines and Control (PEMC) Research Group, Department of Electrical and Electronic Engineering, Faculty of Engineering, University of Nottingham, 15 Triumph Rd, Lenton, Nottingham NG7 2GT, UK; marcoriv@utalca.cl; 4 Laboratorio de Conversión de Energías y Electrónica de Potencia (LCEEP), Faculty of Engineering, Universidad de Talca, Merced 437, Curicó 3341717, Chile; 5 Departamento de Ciencias Agrarias, Campus “San Isidro”, Universidad Católica del Maule, km 6 Camino Los Niches, Curicó 3340000, Chile; miarayaa@ucm.cl

**Keywords:** vine water consumption, crop water stress index, spatial variability, irrigation

## Abstract

The Scholander-type pressure chamber to measure midday stem water potential (MSWP) has been widely used to schedule irrigation in commercial vineyards. However, the limited number of sites that can be evaluated using the pressure chamber makes it difficult to evaluate the spatial variability of vineyard water status. As an alternative, several authors have suggested using the crop water stress index (CWSI) based on low-cost thermal infrared (TIR) sensors to estimate the MSWP. Therefore, this study aimed to develop a low-cost wireless infrared sensor network (WISN) to monitor the spatial variability of MSWPs in a drip-irrigated Cabernet Sauvignon vineyard under two levels of water stress. For this study, the MLX90614 sensor was used to measure canopy temperature (Tc), and thus compute the CWSI. The results indicated that good performance of the MLX90614 infrared thermometers was observed under laboratory and vineyard conditions with root mean square error (RMSE) and mean absolute error (MAE) values being less than 1.0 °C. Finally, a good nonlinear correlation between the MSWP and CWSI (R^2^ = 0.72) was observed, allowing the development of intra-vineyard spatial variability maps of MSWP using the low-cost wireless infrared sensor network.

## 1. Introduction

The adverse effects of climate change will affect the future water availability for agriculture [1,2]. This situation is particularly relevant in viticultural areas of Mediterranean climates, where significant reductions in rainfall are occurring [3]. In commercial vineyards, several irrigation management strategies, such as regulated deficit irrigation (RDI), have been incorporated to overcome the current water shortages [4,5]. The RDI strategy imposes a certain degree of water stress in specific phenological periods where the plant is less susceptible to the lack of water applied during the growing season. RDI has several effects on phenolic compound biosynthesis, yield, and vegetative development depending on the period when water stress is applied [6,7]. However, applying RDI requires careful monitoring of vine water status to avoid significant damage to plant development and reduced yield and fruit quality. 

Several reports have suggested that midday stem water potential (MSWP) can be used as an irrigation tool for monitoring vine water status and thus scheduling irrigation in drip-irrigated vineyards [7,8,9,10]. The MSWP provides significant advantages compared to other irrigation control techniques because it adequately integrates the effect of soil–plant–atmosphere interactions on plant water requirements. However, the MSWP is characterized by low spatial representation at the field level due to a limited number of possible measurements per day. This limitation prevents applying site-specific irrigation management (SSIM) that considers the intra-vineyard spatial variability [11,12,13]. Following this idea, it is essential to note that several spatial scales can contribute to characterizing the spatial variability of different crop characteristics, including plant water status. The most extensive observation scale corresponds to satellite platforms that have allowed the study of plant water status (PWS) over vineyards in large areas [14]. However, the main limitation of satellite-based estimates of PWS is the pixel size or spatial resolution, which makes it very difficult to separate the canopy from the soil surface and shade [15]. Other drawbacks are the temporal frequency of satellite revisit [16] and clouds or other atmospheric disturbances.

The following observation scale is represented by thermal infrared (TIR) sensors mounted on airplanes or unmanned aerial vehicles (UAV) [17]. In this case, the main advantage over satellite platforms is the high spatial resolution, which allows the identification of the canopy due to the pixel size. Additionally, the frequency of visits can be scheduled more efficiently according to critical phenological events in vineyards [18,19,20,21,22,23,24]. However, the main limitation of these technologies is the instrumentation cost [25], flight scheduling logistics [18], limited payload capacity [26], and image processing. The last scale corresponds to the proximal sensing or proxidetection, where the sensors are closer to the object, installed in handheld devices, mounted in fixed locations, or in motorized vehicles. Sensors range from simple RGB cameras to multi-spectral, hyperspectral, and thermal cameras [27]. In this sense, portable infrared radiometers (TIRs) and thermal infrared cameras (TICs) have received much attention in the last two decades due to their ability to assess the plant water status in vineyards growing under different climatic conditions [28]. TIR sensors installed at the field level allow monitoring canopy temperature, which could be related to MSWP measurements by computing thermal-based indices such as the crop water stress index (CWSI). 

The CWSI has been widely used as a crop water status indicator and provides the crop stress level based on canopy–air temperature differences. However, the traditional methodology proposed by [29] is limited by its current cost and the difficulty of implementing spatially distributed sensors to characterize the spatial variability of leaf temperature within the vineyard. To overcome the above, in recent years, the use of wireless infrared sensor networks (WISNs) has emerged, which are presented as an ad hoc network that is autonomous, self-organized, and composed of tens, hundreds, or thousands of smart devices, which are generally battery-powered [30]. These devices have been successfully used for different agricultural applications [31], such as monitoring environmental variables [32,33], microclimatic canopy conditions [26], irrigation management [34], and pest control [35]. These developments include new devices, communication systems, and low-cost sensors to measure leaf temperature and micro-meteorological variables inside the plant canopy. In this regard, the MLX90614 is a low-cost thermal infrared (TIR) sensor that has received special attention due to its accuracy (±0.5 °C), robustness, affordable prices compared to conventional sensors [26], and its possibility of being easily integrated into wireless network systems [26,36,37,38]. For these reasons, some authors have proposed using the MLX90614 as an alternative to the high-cost devices typically used in the field. The cost of a single MLX90614 may represent 1% or even less than a conventional device, making this technology more suited for small- and medium-sized farms that cannot afford to implement a high-cost sensor network. New technologies have also been developed for communication systems to solve the limitations observed when implementing a wireless sensor network at the field level [39]. Due to these technological advances, many research initiatives have been carried out to develop low-cost sensors in agriculture [40,41]. 

Most of these electronic devices have focused mainly on monitoring plant micrometeorological variables (air temperature, wind, and relative humidity), such as in [42], where researchers developed, constructed, tested, and validated a low-cost IoT sensorization node made of open hardware and 3D-printed parts to monitor meteorological variables and detect vineyard diseases. This platform was later strengthened by the same researchers in [43], adding edge computers and a crop model for disease alerts based on the meteorological data provided by the sensors to provide farmers with a decision-support tool to treat downy mildew in vineyards. Other applications consider estimating soil moisture variables to manage RDI [44,45,46]. Broader systems that integrate various devices and computer science solutions have been designed. In this regard, [47] proposed a solution to manage an IoT platform, allowing the user to handle and discover IoT devices for easy use, connection, interoperability, and scalability, handling data management in real-time, considering that one of the most critical challenges is the heterogeneous nature of IoT devices. Despite the above, very few sensors have been developed for monitoring plant water status, considering its spatial variability. 

Therefore, this research presents a wireless infrared sensor network (WISN) to monitor the midday stem water potential (MSWP) in a drip-irrigated Cabernet Sauvignon vineyard. The WISN functions as a self-organized network of devices suitable for agricultural monitoring, including environmental variables and canopy conditions. The MLX90614 sensor was chosen for its cost-effectiveness and relevance to agricultural applications. The research thoroughly examines the WISN’s technical specifications and practical application in precision agriculture.

## 2. Materials and Methods

### 2.1. Experimental Site

**Figure 1:**
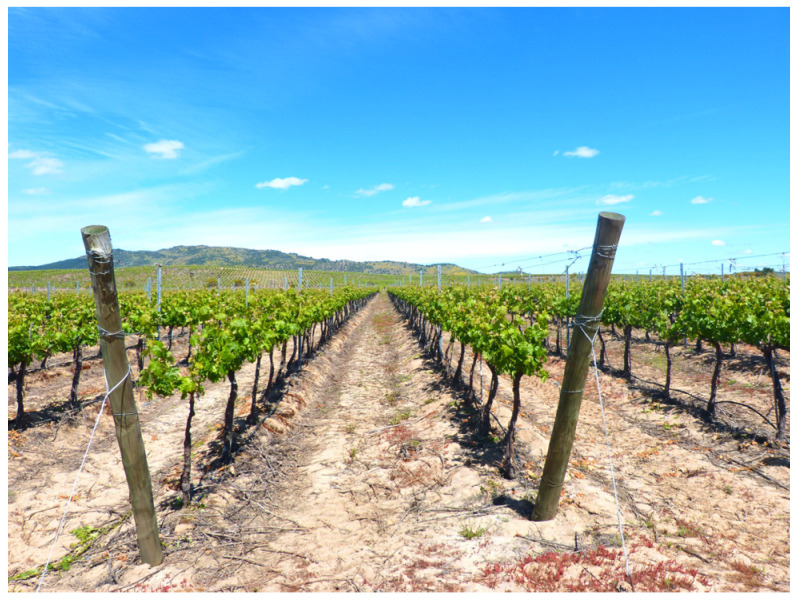
Experimental site.

A WISN was evaluated on a drip-irrigated commercial vineyard (*Vitis vinifera* L., cv Cabernet Sauvignon) located in the Pencahue Valley (Figure 1), Maule Region, Chile (35°20′32.70″ S, 71°46′47.52″ O, 86 m.a.s.l.) during the 2017–2018 growing season (1 September to 30 March). This valley has a Mediterranean climate, with an average temperature of 14.8 °C and accumulated reference evapotranspiration (ETo) of 968.2 mm from September to March. The average annual rainfall in the region is approximately 602 mm, distributed mainly during winter. The summer period is generally hot and dry, with high atmospheric demand. The vineyard’s soil is classified as Quepo series with a clay loam texture. The vineyard was established in 2015, with 2.5 m × 1.5 m spacing trained on a vertical shoot-positioned system (VSP).

### 2.2. Experimental Design

The experimental design consisted of two different irrigation regimes, with four repetitions each, applied after fruit set until veraison to induce different water stress levels over the experimental plot. The treatments were managed according to two MSWP thresholds: no water stress (T1, MSWP > −1 MPa) and moderate–severe water stress (T2, MSWP < −1 MPa). Each treatment was randomly distributed over the experimental plot. Each replicate had six vines, and two central vines were selected for measurements.

### 2.3. Measurement of Meteorological Variables

An automatic weather station (AWS) was installed over the experimental plot spanning 1.4 ha, recording micrometeorological variables at 30-minute intervals. The Vaisala probe (HMP45C Campbell Scientific, Inc., Logan, UT, USA) was employed to monitor the air temperature (Ta) and relative humidity (RH). This probe has a measurement range of 0.8% to 100% RH (non-condensing) for relative humidity and −39.2 °C to +60 °C for temperature. Its accuracy stands at ±1% RH against the factory reference for humidity and ±0.2 °C (up to 20 °C) and ±0.3 °C (up to 40 °C) for temperature. The wind’s speed (u) and direction (w) were captured using an anemometer (YOUNG, 03101-5, Traverse City, MI, USA). This anemometer can measure wind speeds ranging from 0 to 50 m/s (112 mph) with an accuracy of ±0.5 m/s (1.1 mph). Precipitation (Pp) was gauged with a pluviometer (A730RAIN, Adcon Telemetry, Klosterneuburg, Austria). Its accuracy is up to 1% for measurements up to 50 mm and 3% for measurements up to 100 mm. Lastly, the solar radiation (Rs) was quantified with a silicon pyranometer (LI200X, Campbell Scientific, Inc., Logan, UT, USA). This pyranometer has a spectral range of 400 to 1100 nm. In natural daylight, its absolute error is a maximum of ±5%, although ±3% is typical. All of these sensors, responsible for measuring u, w, Pp, Ta, RH, and Rs, were strategically positioned 1.9 m above the vineyard to ensure optimal data capture.

### 2.4. Description of the Low-Cost Wireless Infrared Sensor Network

A network was developed to integrate an infrared thermometer MLX90614 (Melexis, Ypres, Belgium) in eight nodes. This sensor was chosen because of its low cost (10 USD), narrow field of view (FOV) (restricted to 10° by the manufacturer), and capability for noncontact radiometric surface temperature measurements. The sensor package includes a longwave filter that passes radiation from 5.5 to 14 µm. Sensor voltage outputs were converted to temperature readings using the equation Ti = Vj × 0.02 in K and later converted to °C. The manufacturer calibrated this over a wide temperature range, between −40 and 85 °C for ambient temperature and between −70 and 382 °C for object temperature. The standard precision is 0.5 °C relative to room temperature, although medical versions offer a resolution of 0.1 °C at temperatures between 35 and 38 °C. However, it is essential to consider that the thermometer’s accuracy can be influenced by thermal gradients induced across the sensor package [48]. These characteristics make this technology better suited for small- to medium-sized farms that cannot finance costly equipment.

The microcontroller Arduino FIO was used to develop each electronic board. This card was selected due to the following advantages that help reduce the operating cost of the system:

(i) It has a port that connects an Xbee communication card (Xbee S2B).
(ii) It has a port that enables direct connection to a LiPo battery.
(iii) An internal regulator allows attaching a power supply directly to the card.

Finally, all of the components were integrated into a single electronic board.

### 2.5. Wireless Infrared Sensor Network Coordinator Unit

The Arduino Mega microcontroller was used to build the coordinator unit. This device receives and stores information from all nodes installed in the field. The coordinating node comprises an Arduino Mega microcontroller (Atmega2560, Arduino, Somerville, MA, USA) with 54 digital pins, 16 analog inputs, and 4 serial ports for hardware. Its operating frequency is 16 MHz, with a flash memory of 256 KB. Another essential component of the central node is the datalogger, which has a real-time clock (RTC) composed of an integrated circuit DS1307Z, a quartz crystal, and a 3 V CR1220 battery. The data logger also includes a microSD card slot where the information received from the nodes can be stored. The central node communicates with the remote nodes through an Xbee PRO S2B configured to work in a star topology network. Finally, the coordinator unit can send the information received from the nodes via the GPRS-Bee board that provides GPRS and GSM connectivity, thanks to an M95 module. In this way, by request, it sends the information to a database mounted on a virtual private server (VPS). The ABS components were designed and printed on a Creality Ender 3 3D printer to protect the electronic elements under field conditions. The nodes and central units had encapsulations that were adapted to the size of each board.

### 2.6. Data Transmission and Communication Protocols

The system considers two types of communication protocols. It employs Xbee module Series 2B, which uses the IEEE 802.15.4 networking protocol for data transmission from the node to the coordinator unit. Once data are received from the coordinator unit, they are transmitted to the VPS database through GPRS with Wi-Fi technology based on IEEE 802.11 or 3G mobile telecommunication technology protocols. Finally, the data are stored in a MySQL database, which many clients can query to provide data tables, graphical visualization plots, and export data (in .csv format). The user can view data remotely via a browser or a dedicated app.

### 2.7. Calibration and Performance Evaluation of MLX90614 Thermometers

The eight thermometers (MLX90614) mounted on the sensor network were tested in a climatic chamber (CC) under controlled temperature conditions using a blackbody source (Isothermal Technology Limited, Pine Grove, Southport, Merseyside, UK). In the laboratory, each sensor was installed in a fixed position in front of the blackbody at 0.01 m, and then the temperature measured by the sensor was recorded. The blackbody temperatures used in the calibration process ranged from 5 to 65 °C in steps of 5 °C, covering the range of temperatures expected for agricultural applications. The CC temperature was modified from high to low using a cooling/heater system to obtain different ambient temperature values. Therefore, a wide range of temperatures was achieved to evaluate the sensor’s performance.

### 2.8. Leaf Temperature and Midday Stem Water Potential (MSWP)

To measure the MSWP, healthy leaves completely exposed to the sun were selected, first covered with a plastic film and later with an aluminum foil to avoid transpiration, exposure to light, and overheating of the plant tissue. After 1 h, the MSWP measurements were carried out with a Scholander-type pressure chamber. In addition, canopy temperature measurements were performed simultaneously at each node using MLX90614 and MI-2H0 (Apogee Instruments, North Logan, UT, USA) sensors. The WISN was programmed to measure leaf temperature at each node at 15-minute intervals. In contrast, leaf temperature using the MI-2H0 was manually measured along with the MSWP between 1200 and 1500 h (local time).

### 2.9. Crop Water Stress Index (CWSI) Computation

The CWSI was calculated as follows, based on leaf reference methodology (LRM):

(1) $$\mathrm{CWSI}=\frac{T_{c}\;-\;T_{\mathrm{wet}}}{T_{\mathrm{dry}}-T_{\mathrm{wet}}}$$

where $T_{c}$ = canopy temperature (°C), $T_{\mathrm{wet}}\;$= temperature of a fully transpiring leaf (°C), and $T_{\mathrm{dry}}$ = a leaf with fully closed stomata (°C).

This methodology is based on the equation proposed by [49], where $T_{wet}$ and $T_{\mathrm{dry}}$ were obtained from an infrared radiometer, choosing the highest canopy temperature as $T_{\mathrm{dry}}$ and the lowest as $T_{wet}$.

### 2.10. Mapping and Data Processing

Data mapping was performed using QGIS (Version 3.10.13, 2020, QGIS Geographic Information System; Open-Source Geospatial Foundation Project) and 3DField 2.9.0 software. Two quantiles were considered for each water restriction map: (i) low (<1 MPa) and (ii) high water restriction (>1 MPa). Each quantile gathers 50% of the observations. The data were collected on a regular grid for mapping purposes. All statistical analyses were implemented in the R environment.

### 2.11. Statistical Analysis

A sensor evaluation was carried out using linear regression analysis (LRA) through the origin and utilizing root mean square error (RMSE) and mean absolute error (MAE). Additionally, a *t*-test was used to check whether the regression line’s slope (b) significantly differed from unity at the 95% confidence level. Additionally, ANOVA was performed on the entire MSWP database to determine if there were significant differences between both treatments using Fisher’s least significant difference (LSD) test at a confidence interval of 95%.

The LRA, RMSE, and MAE were estimated using the following equations:

(2) $$E_{i}=b*O_{i}$$

(3) $$RMSE=\sqrt{\frac{\sum_{1}^{N}{\left(O_{i}-E_{i}\right)}^{2}}{N}}$$

(4) $$MAE=\frac{1}{N}\sum_{i=1}^{N}\left|E_{i}-O_{i}\right|$$

where $E_{i}$ is the value obtained from MLX90614, O_i_ is the value measured by MI-2H0 or the blackbody, and N is the number of observations. Nonlinear regression models were developed to estimate the MSWP as a function of the CWSI.

Finally, for the analysis of the spatialized maps, the mean values ($\bar{x}$), standard deviation (σ), and coefficient of variation (CV) were used to characterize the spatial variability of the study site.

## 3. Results

### 3.1. Climatic Conditions and Plant Water Status

**Figure 2:**
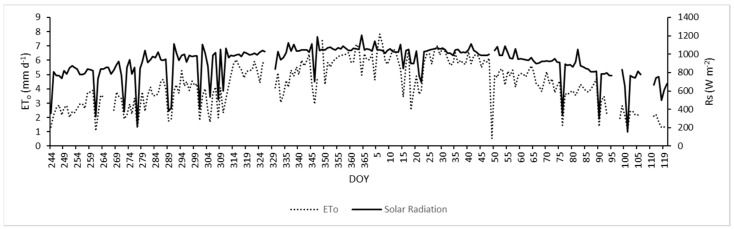
Diurnal variations in reference evapotranspiration (ETo) and solar radiation (Rs) during the 2017–2018 growing season (1 September to 30 March). DOY = day of year.

The diurnal values of ETo and solar radiation (Rs) observed during the 2017–2018 growing season are shown in Figure 2. This figure indicates that the atmospheric conditions were dry and hot during the study period, with maximum values of ETo and Rs recorded between December and January. In this period, the values of ETo ranged between 0.4 and 7.6 mm day^−1^, while those of Rs were between 160 and 1200 W m^−2^. Additionally, rainfall events were scarce, with values less than 10 mm during November and March. In this study, irrigation was applied from 25 October 2017 (DOY 268) to 15 April 2018 (DOY 105).

**Figure 3:**
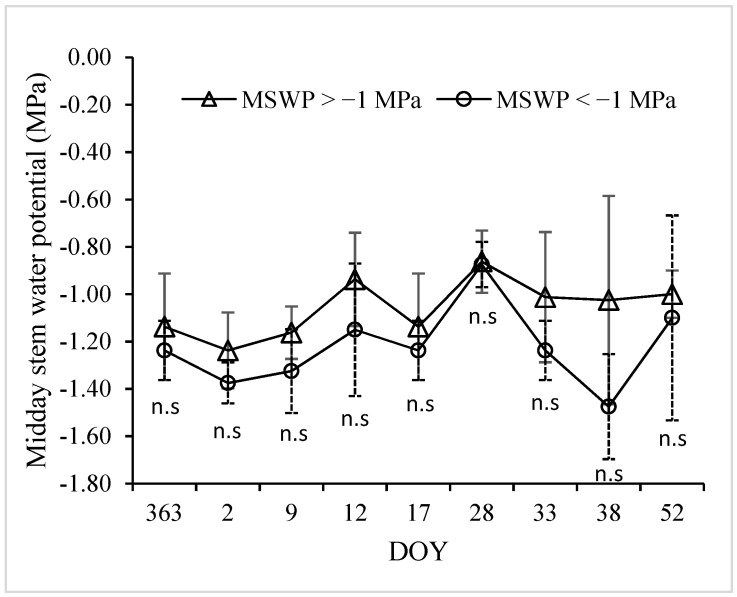
Midday stem water potential (MSWP) measurements for a drip-irrigated vineyard under non and moderate-severe water stress conditions. DOY = day of year.

MSWP measurements were performed simultaneously in each node and irrigation treatment, along with foliage temperature measurements. Figure 3 indicates that the values of MSWP for the T1 treatment ranged between −0.5 and −1.4 MPa, while those for T2 were between −0.8 and −1.65 MPa. These ranges of MSWP were appropriate for developing models for estimating MSWP as a function of the CWSI. Additionally, Figure 3 indicates no significant differences between treatments for the MSWP due to high standard deviations (error bars).

### 3.2. Evaluation of Low-Cost Sensors

**Figure 4:**
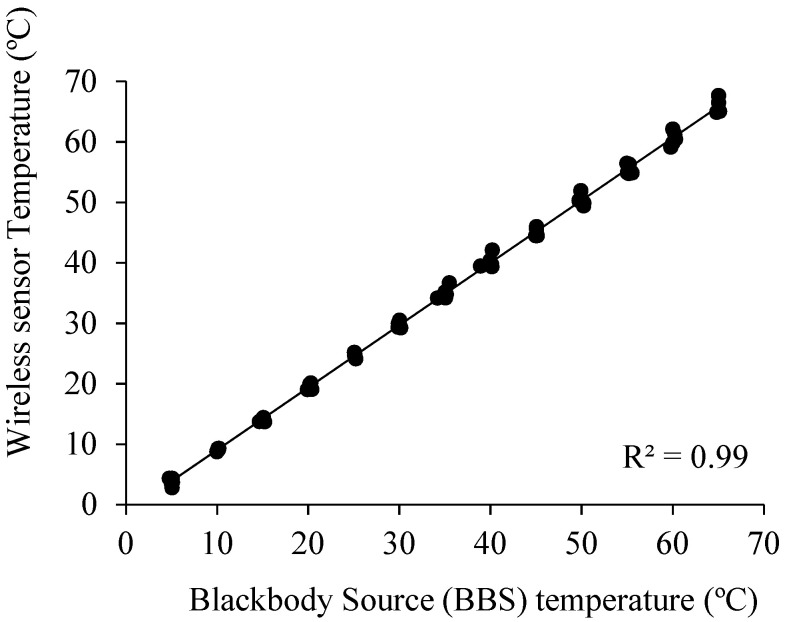
Comparison between temperature measured using a blackbody source (BBS) and obtained using MLX90614 sensors in a climatic chamber under controlled temperature conditions.

The evaluation of the MLX90614 sensors using a blackbody source (BBS) under different temperature ranges (between 5 and 65 °C) is shown in Figure 4. This figure indicates that the MLX90614 sensors presented excellent performance, with R^2^, MAE, and RMSE values equal 0.99, 0.83 °C, and 0.99 °C, respectively (Table 1). In addition, the *t*-test indicated that the b value was statistically equal to one, stating that temperatures from the MLX90614 sensors and BBS were similar at a 95% confidence level. Under vineyard conditions, good agreement between MLX90614 and MI-2H0 was observed for estimating the leaf temperatures of drip-irrigated vines under MSWP ranging between −0.65 MPa and −1.7 MPa. In this case, the R^2^, MAE, and RMSE values were 0.99, 0.83 °C, and 0.99 °C, respectively (Table 1). 

**Figure 5:**
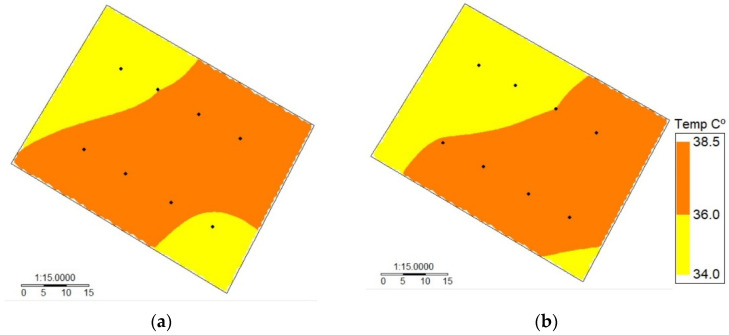
Intra-vineyard spatial variability of leaf temperature measured automatically using (**a**) a wireless infrared sensor network (WISN) and (**b**) manually using an MI-2H0 commercial sensor for a drip-irrigated vineyard under two irrigation treatments (DOY 363).

Additionally, the *t*-test pointed out that leaf temperatures from the MLX90614 and MI-2H0 were similar, demonstrating the robustness of the proposed sensor. However, one MLX90614 sensor presented errors due to a factory defect. This situation shows the importance of evaluating each device before incorporating it into a more complex and sophisticated sensor network, as proposed in this research. This avoids uncertainty in the data collection that could produce an incorrect interpretation of model outputs. As an example, the leaf temperature spatial variability for DOY 363 obtained using the MLX90614 and MI-2H0 is shown in Figure 5. In this case, a similar spatial pattern of leaf temperature for the two sensors was observed within the drip-irrigated vineyard, where the values of Tc ranged between 34.7 and 38.6 °C and 33.5 and 38.1 °C for the WISN and MI-2H0, respectively.

**Figure 6:**
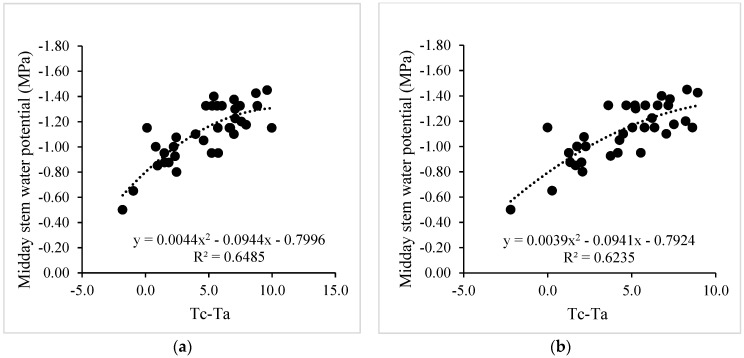
Nonlinear regression analyses between the midday stem water potential (MSWP) and canopy (Tc) and air (Ta) temperature difference. (**a**,**b**) indicate that Tc was obtained using a low-cost wireless infrared sensor network (WISN) and MI-2H0 sensors, respectively.

**Figure 7:**
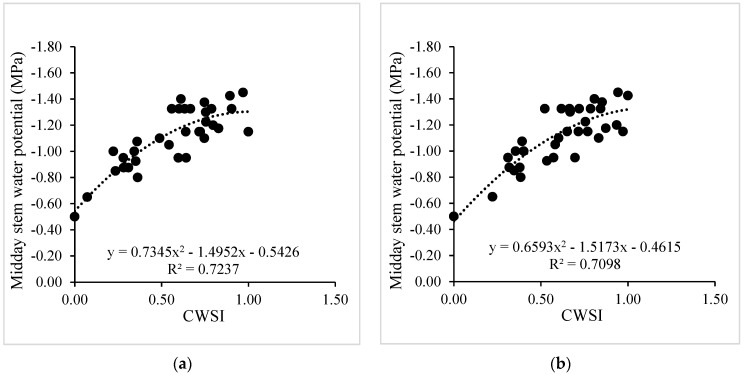
Nonlinear regression analyses between the crop water stress index (CWSI) and midday stem water potential (MSWP) estimated using (**a**) and low-cost wireless infrared sensor network (WISN) and (**b**) MI-2H0 commercial sensors.

**Figure 8:**
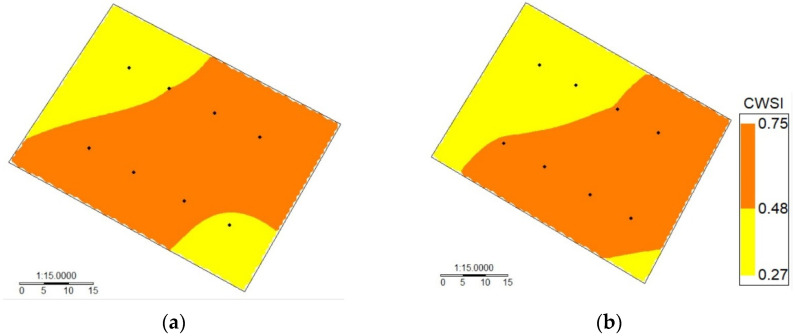
Intra-vineyard spatial variability of the crop water stress index (CWSI) automatically measured using (**a**) a wireless infrared sensor network (WISN) and (**b**) manually using an MI-2H0 commercial sensor for a drip-irrigated vineyard under two irrigation treatments (DOY 363).

After validating the low-cost wireless sensors to measure leaf temperature, a nonlinear regression analysis between the MSWP and canopy–air temperature difference (Tc-Ta) was performed for drip-irrigated vines under two levels of water stress (Figure 6). In this case, the R^2^ values for the nonlinear regression using Tc obtained from the MLX90614 and MI-2H0 sensor were 0.65 and 0.62, respectively. In addition, the MAE and RMSE values for the two TIR sensors were 1.20 and 0.77 °C, respectively. Similarly, the regression between the CWSI and MSWP was significant, with R^2^ values of 0.72 and 0.71 for the WISN and the commercial sensor, respectively (Figure 7). Additionally, the MAE and RMSE values for the CWSI estimations were 0.11 and 0.08, respectively. The intra-vineyard spatial variability of the CWSI using the WISN and commercial sensors for vine grapes is shown in Figure 8. In this case, the WISN adequately described the spatial variability behavior of the three levels of the MSWP.

**Figure 9:**
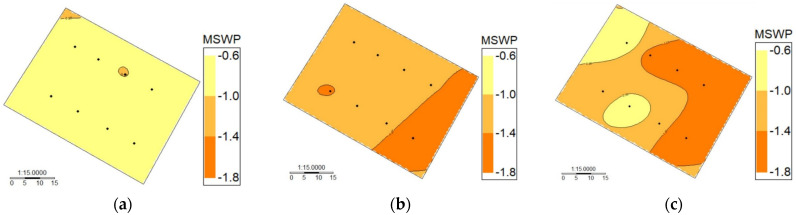
Intra-vineyard spatial variability of midday stem water potential (MSWP) automatically estimated using a wireless infrared sensor network (WISN) for a drip-irrigated vineyard under different levels of water status. (**a**): Day of year (DOY) 28, (**b**): day of year (DOY) 2, and (**c**): day of year (DOY) 38.

Figure 9 shows the intra-vineyard spatial variability of MSWP using the WISN for the drip-irrigated vineyard under different levels of water status. In this case, days were selected according to Figure 3, which indicates that the mean values of the MSWP were −1.31 MPa for DOY 2, −0.87 MPa for DOY 28, and −1.25 MPa for DOY 38. The lowest spatial variability was observed on DOY 28 (Figure 9a), which presented a standard deviation (SD) and variation coefficient (CV) of 0.11 MPa and 12.28%, respectively. Additionally, on this day, a small “artifact” was observed in the upper left corner due to the spatial interpolation method. The highest spatial variability was found on DOY 38 (Figure 9c), with a wide range of MSWP ranging from −0.65 to −1.7 MPa. Three clusters were identified for this day, with SD and CV values of 0.4 MPa and 32.21%, respectively. DOY 2 presented two clusters with an SD and CV of 0.14 and 10.71%, respectively, indicating medium spatial variability.

## 4. Discussion

### 4.1. A Brief Comparison with the Current State of Technology in Precision Agriculture

Precision agriculture has witnessed a rapid evolution over the past few decades, with technological advancements playing a pivotal role in shaping its trajectory. Several methodologies and tools have been developed to optimize agricultural practices, particularly in vineyard management.

- Wireless sensor systems: These systems have emerged as a fundamental part in precision agriculture. Their ability to provide real-time monitoring of crop and soil conditions has allowed informed agricultural decisions to be made [50,51,52]. Energy efficiency in these networks is also a significant research topic, ensuring sustainable and long-term monitoring [53].
- Thermal imaging: This technology has been extensively used to assess water status within vineyards. Recent advancements have enabled high-resolution thermal imagery to estimate the variability of plant water status within vineyards [54,55,56]. Ground-based thermal imaging has also assessed crop water status across different growth stages [57].
- Geospatial analysis tools: Tools such as QGIS have been highlighted for their ability to interpret and visualize data, providing a greater understanding of spatial patterns within vineyards. These tools have transformed how data are processed and understood in the agricultural domain.
- Wireless communications: The ZigBee wireless protocol has been emphasized for ensuring a seamless and consistent flow of information [58]. This continuous data stream is crucial for real-time decision-making in precision agriculture.
- Irrigation management: Various studies have explored the intricacies of irrigation management, emphasizing the importance of real-time monitoring and data-driven decision making [59].
- Machine learning algorithms: Integrating machine learning with remote sensing data has paved the way for more sophisticated and accurate irrigation scheduling management [60,61].

Compared to the existing methodologies, our study integrates the strengths of wireless sensor systems and geospatial analysis tools to provide a comprehensive solution for vineyard management. Our approach emphasizes real-time monitoring, efficient data processing, and informed decision-making, ensuring optimal resource allocation and sustainable agricultural practices.

### 4.2. Communications and Power Consumption

After analyzing the results obtained in this study, we believe that to obtain a constant and effective flow of information, consideration must be given to wireless communications; in our case, we used the ZigBee wireless protocol, which is considered one of the best candidate technologies for agricultural applications [53]. Some of the most important advantages of this protocol correspond to its ability to conserve energy by switching between active and sleep states, which is why power consumption can be minimized, lengthening the useful life of the sensor’s battery. On the other hand, this system allows the establishment of different types of communication in a simple way, which allows any user with a medium level of training to program a network. Therefore, it was decided to integrate this communication protocol into the sensor developed to allow the system to establish communication between its components simultaneously. The Xbee S2B module was used in this research due to its adequate range/price ratio. Numerous instances of using these devices can be found in the literature, especially in precision agricultural applications [62]. Despite the positive comments reported in the literature, in this particular case, it is essential to point out that Xbee S2B may significantly vary its communication range due to local operating conditions [63]; thus, for optimal communication, the antennas must have a direct line of sight to each other; otherwise, the range can be reduced up to 30% according to the communication tests carried out in this investigation, being significantly affected by the metallic structure of the vineyard, as well as the plant’s canopy.

In addition, the reader should consider that due to the rapid advancement of technology, there are other alternatives for establishing wireless communication systems. An example of this is narrowband (NB) IoT technologies, such as long-range radio (LoRa); due to low power consumption, it is preferably used when agricultural information is to be transmitted over long distances [53]. This technology offers the opportunity to improve this sensor or other future developments by integrating this receiver, which costs a fraction of ZigBee antennas. Regarding GPRS communication, it is important to point out that during the implementation of this study, high intermittence was generated in sending data, with the system being disconnected from the network most of the time. This was due to the low coverage of telephone companies in rural sectors, which has systematically hindered the implementation of these technologies at the field level in central Chile. Therefore, it is suggested to resort to other technologies and network topologies that allow direct access to local routers that upload the information through home networks with a much more stable connection over time to constantly flow information without interruptions. Current consumption is another critical factor reported in the literature for developing wireless sensor networks. In this sense, we suggest using simple low-consumption microcontrollers to prioritize the device’s energy efficiency.

In the same way, we suggest (whenever possible) that the integration of solar charging systems significantly increases the autonomy of the devices [64]. However, care is recommended when sizing the device’s electrical needs because solar energy is not constant and is intermittent at the field level. If the foregoing is rigorously evaluated, solar energy is an attractive alternative to provide autonomy to sensors deployed in remote field sectors where access is difficult.

### 4.3. Stress Index Computations

This study demonstrates that the WISN could automatically simulate the intra-vineyard spatial water status variability for drip-irrigated vines growing under MSWP ranging from −0.5 to −1.65 MPa. In this case, the low-cost thermal infrared sensors (MLX90614) responded adequately to different levels of MSWP, with Tc ranging between 25.79 and 37.91 °C. A significant nonlinear correlation between MSWP and CWSI was observed with an R^2^ = 0.72. These results agree with those in the literature. In this regard, [65] developed a CWSI-based model that computed MSWP with an R^2^ = 0.78 when using infrared thermometers (SI-1H1, Apogee Instruments) installed in a drip-irrigated vineyard. Using thermal infrared cameras (TICs), [57,66,67] estimated plant water status with R^2^ values ranging between 0.58 and 0.67. However, the most common portable thermal sensors (nonthermal-compensated microbolometers) are highly sensitive to variations in ambient temperature and, therefore, must be carefully calibrated and managed to obtain accurate determinations at the field level. The above applies to handheld devices and devices mounted on unmanned aerial vehicles (UAVs). Some authors, such as [68,69], explored the use of thermal infrared sensors (model PC151LT-0, Calex Electronics) deployed at the field level and TICs (FLIR SC655, FLIR Systems, Portland, OR, USA) placed on a UAV to monitor the plant water status spatial variability in a commercial vineyard, observing R^2^ values ranging between 0.46 and 0.91 for the leaf water potential (LWP)–CWSI and MSWP–CWSI correlations, respectively. Considering the above, we can point out that the results obtained in this research are consistent with the data reported by other authors in the literature.

### 4.4. Final Considerations

Finally, it is essential to highlight that crop monitoring requires sophisticated approaches to integrate information across temporal and spatial scales. Technological advances in automated data collection have enabled higher spatial, spectral, and temporal resolutions at a geometrically declining cost per unit area [70]. Satellite and airborne sensors are also helpful in observing large areas; however, for massive use, their high cost still needs to be improved, and there is a need to have specialized personnel for their operation. For this reason, despite the difficulties in implementing low-cost devices (reduction in the Xbee operating range in field conditions, intermittence of GPRS connectivity, and current consumption), their application on a farm scale could address the problem of cost and allow consideration of spatial variability, making this solution more attractive than conventional remote sensing techniques.

## 5. Conclusions

This study assessed the effectiveness of a spatialized wireless sensor network using low-cost thermal infrared (TIR, MLX90614) technology to monitor the spatial variability of MSWP in drip-irrigated vineyards under varying levels of water stress. With the technology provided, areas of water stress could be precisely identified, allowing for timely interventions. One of the most notable findings from the research was the transformative potential of wireless sensor systems in the agricultural landscape. The MLX90614 infrared thermometers performed well under laboratory and vineyard conditions, with MAE and RMSE values less than 1.0 °C. Furthermore, the correlation between MSWP and CWSI (R^2^ = 0.72) enabled the development of intra-vineyard spatial variability maps of MSWP using the low-cost wireless infrared sensor network, enabling the identification of differentiated management zones. Moreover, this study emphasized the significance of wireless communications in agriculture. The ZigBee wireless protocol was highlighted for its ability to ensure a consistent and effective flow of information. This continuous data stream is essential in precision agriculture, where real-time decisions can profoundly impact crop health and yield. As evidenced by the study, these systems have proven to be instrumental in real-time monitoring of crop and soil conditions. Their ability to gather data efficiently and consistently has paved the way for more informed decision-making processes, ensuring that agricultural practices are both sustainable and efficient. Finally, the research underscored the importance of geospatial analysis tools in interpreting and visualizing the collected data. Tools such as QGIS have transformed how data are processed and understood, allowing for a more nuanced understanding of spatial patterns within vineyards. This has facilitated more informed decision-making processes, ensuring that resources are allocated optimally, and interventions are carried out where they are most needed.

## Figures and Tables

**Table 1:** Evaluation of surface temperature using MLX90614 under controlled and vineyard conditions.

Deviance Measures	Controlled Conditions	Vineyard Conditions
MAE (°C)	0.26	0.83
RMSE (°C)	0.28	0.99
b	0.99	0.98
R^2^	0.99	0.99
*t*-test	T	T

MAE = mean absolute error; RMSE = root mean square error; and b = ratio of observed to computed values. R^2^ = coefficient of determination, T null hypothesis (b = 1) true, and F alternative hypothesis (b ≠ 1) false.

## Data Availability

Data are contained within the article.
